# Spectrum and Risk of Neoplasia in Werner Syndrome: A Systematic Review

**DOI:** 10.1371/journal.pone.0059709

**Published:** 2013-04-01

**Authors:** Julia M. Lauper, Alison Krause, Thomas L. Vaughan, Raymond J. Monnat

**Affiliations:** 1 Department of Epidemiology, University of Washington, Seattle, Washington, United States of America; 2 Department of Pathology, University of Washington, Seattle, Washington, United States of America; 3 Department of The School of Medicine, University of Washington, Seattle, Washington, United States of America; 4 Fred Hutchinson Cancer Research Center, Epidemiology Program, Public Health Sciences Division, Seattle, Washington, United States of America; 5 Department of Genome Sciences, University of Washington, Seattle, Washington, United States of America; Albert Einstein College of Medicine, United States of America

## Abstract

**Background:**

Werner syndrome (WS) is an autosomal recessive genetic instability and progeroid (‘premature aging’) syndrome which is associated with an elevated risk of cancer.

**Objectives:**

Our study objectives were to characterize the spectrum of neoplasia in WS using a well-documented study population, and to estimate the type-specific risk of neoplasia in WS relative to the general population.

**Methods:**

We obtained case reports of neoplasms in WS patients through examining previous case series and reviews of WS, as well as through database searching in PubMed, Google Scholar, and J-EAST, a search engine for articles from Japan. We defined the spectrum (types and sites) of neoplasia in WS using all case reports, and were able to determine neoplasm type-specific risk in Japan WS patients by calculating standardized incidence and proportionate incidence ratios (SIR and SPIR, respectively) relative to Osaka Japan prefecture incidence rates.

**Results:**

We used a newly assembled study population of 189 WS patients with 248 neoplasms to define the spectrum of neoplasia in WS. The most frequent neoplasms in WS patients, representing 2/3 of all reports, were thyroid neoplasms, malignant melanoma, meningioma, soft tissue sarcomas, leukemia and pre-leukemic conditions of the bone marrow, and primary bone neoplasms. Cancer risk defined by SIRs was significantly elevated in Japan-resident WS patients for the six most frequent neoplasms except leukemia, ranging from 53.5-fold for melanoma of the skin (95% CI: 24.5, 101.6) to 8.9 (95% CI: 4.9, 15.0) for thyroid neoplasms. Cancer risk as defined by SPIR was also significantly elevated for the most common malignancies except leukemia.

**Conclusions:**

WS confers a strong predisposition to several specific types of neoplasia. These results serve as a guide for WS clinical care, and for additional analyses to define the mechanistic basis for cancer in WS and the general population.

## Introduction

Werner syndrome (WS, OMIM #277700) is an autosomal recessive disease caused by loss of function mutations in the *WRN* gene. WS patients develop features reminiscent of premature aging beginning in the second decade of life, including bilateral cataracts, graying and loss of hair, scleroderma-like skin changes, diabetes mellitus, and osteoporosis (Table 1) [1]. WS patients are also at elevated risk for common, clinically important age-dependent diseases such as cancer and atherosclerotic cardiovascular disease which are the most common causes of death at a median age of 54 years [1]–[3].

**Table 1:** Werner syndrome diagnostic criteria.

Category	WS signs and symptoms*
Cardinal**	1.Cataracts (bilateral)
	2.Characteristic dermatological pathology
	3.Short stature
	4.Parental consanguinity
	5.Premature greying and/or thinning of scalp hair
Additional	1.Diabetes mellitus
	2.Hypogonadism
	3.Osteoporosis
	4.Osteosclerosis of distal phalanges of fingers and/or toes
	5.Soft tissue calcification
	6.Premature atherosclerosis
	7.Mesenchymal neoplasms, rare neoplasms or multiple neoplasms
	8.Voice changes
	9.Flat feet

* WS signs and symptoms are from the diagnostic criteria established by the International Registry of Werner Syndrome: www.wernersyndrome.org/registry/diagnostic.html.

** Reported cataracts were assumed bilateral if not explicitly stated, and characteristic dermatological pathology was considered to be present if any one of the six skin pathologies was reported, again as defined by the Werner Syndrome Registry diagnostic signs and symptoms list. Where height was not designated as ‘short stature’, we classified males with heights <164 cm and females with heights <154 cm as of short stature. Diabetes mellitus was considered present if a diagnosis of diabetes and/or evidence of abnormal glucose homeostasis was provided.

**Figure 1:**
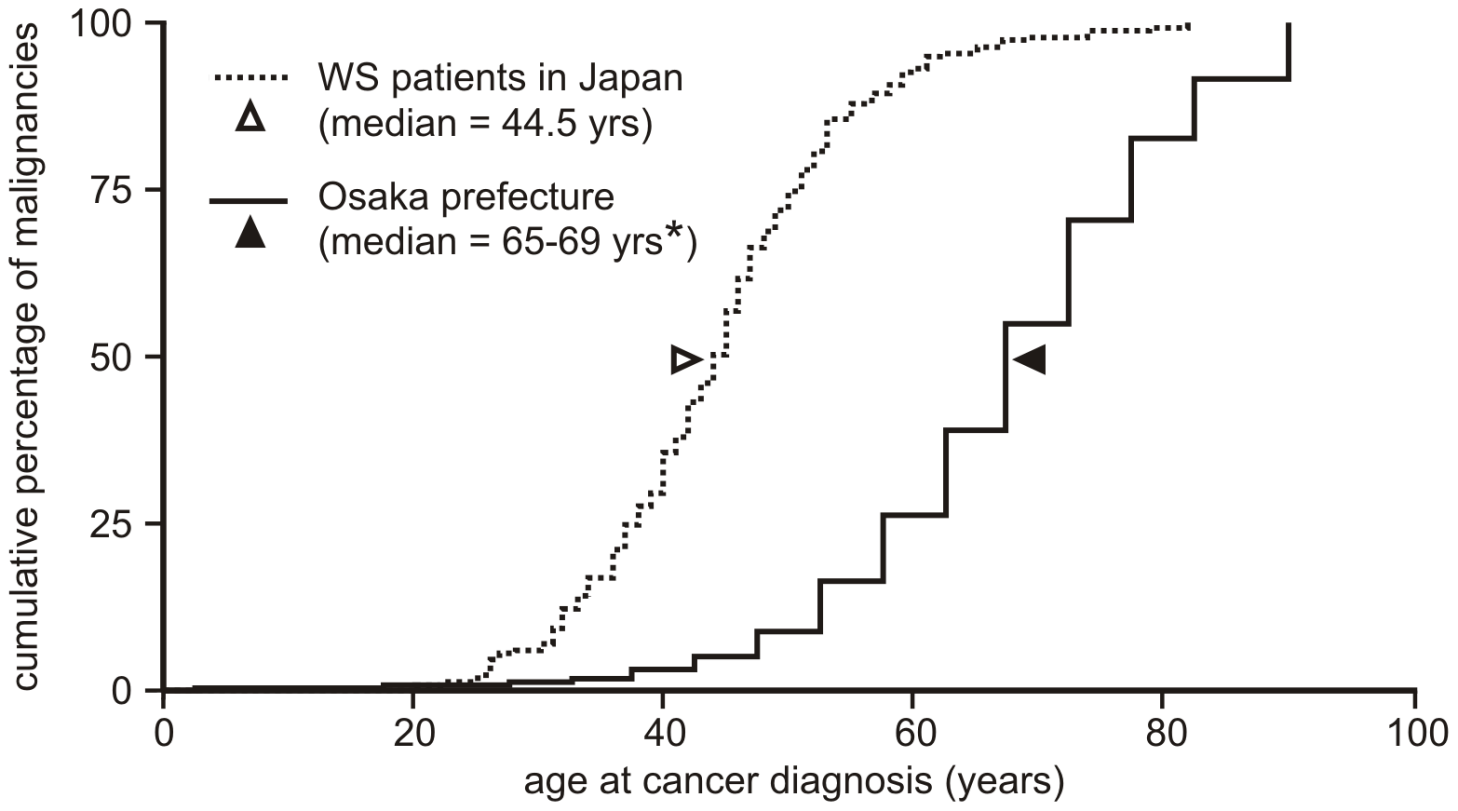
Cumulative percentage of malignancies (%) by age at diagnosis in Japan-resident WS cases (1965–2009) versus Osaka population (1998–2002). *Osaka prefecture neoplasm-specific population incidence data were categorized by gender and aggregated into 5-year age groups. Hence we were able to calculate only a median age range from Osaka population data, in contrast to the exact median age we were able to calculate for our WS patient cohort.

Both the high frequency and additional features of cancer in WS support the idea that WS is a cancer predisposition syndrome. These features include: early age of onset; a high frequency of specific tumor types, including uncommon tumor types; unusual tumor sites; and the presence of multiple, often histologically distinct tumors in individual patients [1], [4]–[6] (Figure 1).

In order to better define the spectrum (types and sites) and provide the first rigorous estimates of type-specific increased risk of cancer in WS, we assembled a carefully documented worldwide study population of WS patients with cancer. We used the largest subset of this study population–WS patients residing in Japan–to estimate neoplasm type-specific risk and the overall risk of neoplasia relative to the Osaka prefecture population. Our results provide the first detailed and quantitatively rigorous view of cancer type and associated type-specific risk in WS.

## Materials and Methods

### WS Case-finding

We used systematic searches to identify all case reports of neoplasia in WS in English or other languages including Japanese (see Methods S1). We followed PRISMA guidelines for conducting and reporting these searches (see Figures S1 and S2 for the PRISMA checklist and flow diagram). Case reports of WS individuals with neoplasia from Japan were identified by searching J-EAST (http://sciencelinks.jp/j-east/). Additional case reports from Japan were identified by searching PubMed (http://www.ncbi.nlm.nih.gov/pubmed/). To identify WS patients with neoplasms residing outside of Japan, we conducted searches in PubMed and Google Scholar (http://scholar.google.com/). Case reports were reviewed in full if they referred to WS and noted a tumor or neoplasm in the title or abstract. For completeness we also reviewed additional WS case reports that did not specifically refer to a ‘tumor,’ ‘neoplasm’ or ‘cancer’ in the title or abstract. The reports assembled from these searches were cross-correlated with previously published case reports or series (see, e.g., Goto *et al*. 1996) [5] to identify and remove duplicate reporting (Table S1). Less well-documented reports of neoplasia in WS were not included in analyses, but were cataloged for future reference and analysis (Table S2). Searches were completed on 1 September 2011.

We confirmed the diagnosis of Werner syndrome in our newly assembled case population using standard diagnostic criteria established by the International Registry of Werner Syndrome (http://www.wernersyndrome.org/registry/diagnostic.html) (Table 1), and assigned all patients a WS diagnostic confidence of ‘definite’, ‘probable’, ‘possible’ or ‘uncertain’ (Tables 2 and 3). We collected additional demographic, clinical and tumor-specific information on all cases (see Methods S1). Tumor diagnoses were verified by comparing the reported diagnosis with patient-specific gross pathology and histopathology images whenever available.

**Table 2:** Werner syndrome diagnostic confidence categories.

*Diagnostic category*	*Sensitivity analysis category*	*Diagnostic criteria*
Definite	High confidence	all cardinal signs + two additional signs *OR* confirmed pathogenic mutations in both *WRN* alleles
Probable	High confidence	first 3 cardinal signs + any 2 others
Possible	Low confidence	either cataracts or dermatological changes + any 4 additional signs
Exclusion**	Exclude	Onset of signs or symptoms before adolescence (except stature)

***Diagnostic criteria and categorization notes:***

* WS diagnostic confidence categories were taken from the International Registry of Werner Syndrome: www.wernersyndrome.org/registry/diagnostic.html with the following modifications: 1. putative WS patients with known pathogenic mutations in both *WRN* alleles were also considered to be ‘Definite’/‘High confidence’; and 2. “mesenchymal neoplasms, rare neoplasms or multiple neoplasm” was not counted for any of the patients in the determination of Werner syndrome diagnostic confidence.

** We had no case exclusions based on the onset of signs or symptoms prior to adolescence. Two Japan-resident cases were reported with voice changes prior to adolescence, and one Japan non-resident case was reported to have had premature greying of the hair at age 8 (see Table S1).

**Table 3:** Werner syndrome study population.

WS patient type	patients (n)	gender (M/F/ns*)	mean age atfirst neoplasmdiagnosis(in yrs.)**	age at first neoplasmdiagnosis(range, in yrs.)**	neoplasms (n)	number/% multipleneoplasm patients
residing in Japan***	139	79/58/2	43.9	20–69	184	32 (23%)
residing outside Japan****	50	23/26/1	41.4	23–56	64	10 (20%)
**Total**	**189**	**102/84/3**	**43.3**	**20–69**	**248**	**42 (22%)**

* ns = not specified or reported.

** among patients whose age at diagnosis is specified.

*** number of patients by WS diagnostic confidence: definite (n = 50), probable (n = 35), possible (n = 22), and unknown (n = 32).

**** number of patients by WS diagnostic confidence: definite (n = 22), probable (n = 13), possible (n = 10), and unknown (n = 5).

### Population Comparison Data

Population-based cancer incidence data were obtained from WHO *Cancer Incidence in Five Continents* (*CI5*) data available online [7]. Japanese population comparison data for risk estimation was from the Osaka prefecture, which has both a large population and a long-established cancer registry. Population incidence data on benign meningiomas, which are not covered in WHO *CI5* data, were provided by the Osaka Cancer Registry as de-identified individual records of benign and malignant meningiomas (ICD-10 codes C70.0–70.9, D32.0–32.9, D42.0–42.9) categorized by gender, age and year at diagnosis. Similar case data on malignant cutaneous melanoma (ICD-10 code C43.0–43.9) were obtained to assess the relative risk for acral lentiginous melanoma (ICD-O-3M code 8744/3).

### Ethics Statement

Case report and population comparison data were publicly available and/or de-identified, and our data collection and analysis plan did not require IRB approval as assessed by the University of Washington Human Subjects Division (reference #42092) and the Ethical Committee of Osaka Medical Center for Cancer and Cardiovascular Diseases (request #11-0006 for Osaka Cancer Registry data).

### Spectrum of Neoplasia: Histopathologic and Geographic Analyses

Neoplasms were classified by histopathologic type and site, with reinterpretation or translation to match current diagnostic classification criteria and WHO International Classification of Diseases for Oncology (ICD-O) nomenclature and guidelines [8]. Cancers or malignancies not otherwise specified were assumed to be carcinomas at the following organ sites: breast, larynx, esophagus, ovary, thyroid, uterus, nasal cavity and liver. Unspecified stomach and pancreas malignancies were assumed to be adenocarcinomas. The most frequent neoplasms by type and site were identified using all study population case data included in Table S1. For risk estimation these data were subdivided into neoplasms arising in Japan-resident patients (virtually all of whom were of Japanese descent), and in patients residing outside of Japan regardless of ethnicity for risk analyses.

We tested whether the frequencies of soft tissue sarcomas, bone neoplasms, malignant melanomas, meningiomas, hematologic/lymphoid neoplasms, thyroid neoplasms, or all other neoplasms differed significantly between Japan-resident patients and WS patients residing outside Japan. Local Fisher’s exact tests were performed for each neoplasm type or group, and a global Fisher’s exact test was performed to detect overall differences in the distribution of neoplasms between patient subgroups (see Statistical Methods below for additional detail).

We also compared the frequency of thyroid malignancy subtypes in Japan-resident WS patients to Osaka prefecture comparison data from *CI5* (*CI5* volumes 7–9, years 1988–2002, available from http://ci5.iarc.fr/CI5i-ix/ci5i-ix.htm) [9]–[11], using Fisher’s exact test. Thyroid malignancies were classified as papillary, follicular, or other/unspecified subtype for this analysis.

### Relative Risk Analyses

We calculated neoplasm-specific standardized proportionate incidence ratios (SPIRs) to determine the extent to which the most frequently reported neoplasms in WS were over-represented in Japan-resident WS patients versus a Japan reference population. The proportions of specific malignant neoplasms observed in WS patients between 1965–2009 were compared to the proportions expected based on *CI5* data for the Osaka prefecture between 1970–2002, adjusted for age (10–39 years and 40–69 years), time period (prior to 1988, and ≥1988), and gender. SPIRs were calculated for soft tissue and connective tissue malignancies (ICD-10 C47 & C49); bone malignancies (ICD-10 C40–41); malignant melanomas of the skin (ICD-10 C43); leukemias (ICD-10 C91–95); and thyroid malignancies (ICD-10 C73).

Standardized incidence ratios (SIRs) were calculated to estimate neoplasm-specific relative risk for all malignancies and meningiomas (ICD-10 codes C70.0–70.9, D32.0–32.9, D42.0–42.9). We estimated the SIR by comparing WS cases to the expected number calculated from gender- and age-specific population incidence rates and the estimated number of WS patients in Japan. Patient numbers were estimated from pathogenic *WRN* allele frequency data using the Hardy-Weinberg equilibrium. Japan population data were used to estimate the age distribution of WS patients at risk, and the estimated age distribution was adjusted to account for the shortened life expectancy in WS (median age at death of 54.3 years) [2], [3]. Osaka prefecture neoplasm-specific population incidence data were obtained from *CI5* data, in which annual cases were categorized by gender and divided into 5-year age groups. The expected number of cases was calculated using indirect standardization by applying the population incidence rates to the estimated WS patient population, with rates adjusted for gender, 10-year age groupings from ages 10–69 (population incidence rates for ages 60–69 were applied to WS patients estimated to be ages 60+) and time period (2–6 year intervals from 1965–2009, based on *CI5* periods).

Exact 95% confidence intervals were obtained for each risk estimate. For sensitivity analyses, we re-calculated the WS patient population at risk based on upper (q = 0.006) [12] and lower (q = 0.0014) [13] *WRN* pathogenic allele frequency estimates. SIR and SPIR analyses were conducted using Japan-resident WS cases with the highest confidence (‘definite’ or ‘probable’) WS diagnoses only. Additional detail for both the SPIR and SIR analyses can be found in Methods S1.

### Statistical Methods

Analyses were performed using Stata 12.0 (StataCorp, College Station, TX, USA). Fisher’s exact tests were two-sided; for neoplasm-specific tests of geographic variation in tumor type, a Bonferroni correction was applied to the α = 0.05 threshold for statistical significance. In both SPIR and SIR analyses, the exact 95% confidence interval for each estimate was obtained using the Stata “istdize” function.

## Results

Database searches and reviews of prior case series (see Figure S2) identified 248 full-text articles for review. From these full-text articles, we identified 202 articles describing one or more neoplasms in a WS patient. After applying the exclusion criteria to tumor reports in these articles (see Methods S1), our final study population consisted of 189 well-documented Werner syndrome (WS) patients with 248 neoplasms. This population included 139 Japan-resident patients, and an additional 50 patients of diverse ethnicities and locations outside of Japan that were reported between 1939 and 2011 (Table 3). The mean age at first diagnosis of neoplasia was 43.3 years ±9.9 years (range 20–69) (Table S1), and the distribution of ages at cancer diagnosis is left-shifted (advanced) by ∼20 yrs compared with the general Osaka population (Figure 1). An additional 87 tumor reports that did not meet our study population inclusion criteria were also cataloged for completeness though not further analyzed (Table S2).

### Spectrum of Neoplasia in WS

**Figure 2:**
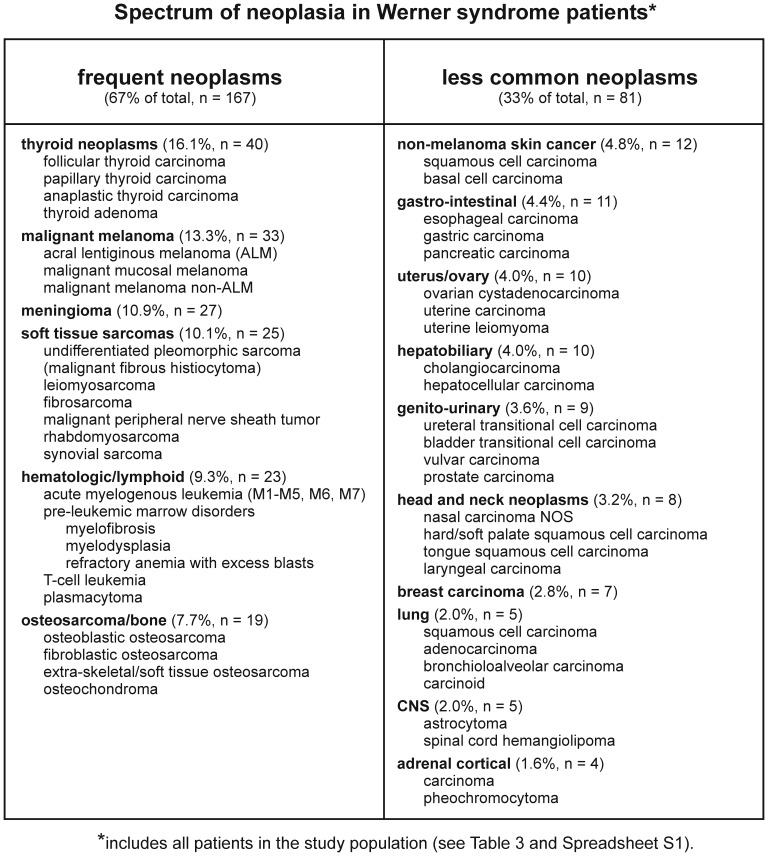
Spectrum of neoplasia in Werner syndrome patients. Distribution of neoplasms by histopathologic type and frequency among study population subjects included in Table S1.

Two-thirds (67%) of all reports of neoplasia in our WS patient population were of 6 different neoplasms: thyroid neoplasms, malignant melanoma, meningioma, soft tissue sarcomas, leukemia and pre-leukemic conditions and osteosarcoma/bone neoplasms (Figure 2). Malignant melanomas were almost exclusively less common variants: acral lentiginous melanomas arising on the palms, soles or in nail beds; and mucosal melanomas arising in the nasal cavity or esophagus. Thyroid neoplasms and melanomas were less frequent in WS patients residing outside Japan, although these differences were not statistically significant after correcting for multiple testing (thyroid: p = 0.011; melanoma: p = 0.057; Table S3). Thyroid neoplasms in Japan-resident patients included a disproportionately high number of follicular carcinomas compared to population control data (p = 0.00002, using reference population ages 0–24, and p = 0.00001 for reference population ages 10–69; Table S4). Leukemias covered the full spectrum of morphologies, though we lacked data to assign a WHO and/or FAB classification to all cases. Atypical leukemias and a wide range of preleukemic disorders were also reported, including myelodysplasia, myelofibrosis and refractory anemia with an excess of blasts (RAEB) (Figure 2). Multiple neoplasms were common: 22% of WS patients (42/189) had 1 to 4 additional, concurrent or sequential neoplasms. These were often at different sites, and of substantially different type(s) (Table S5).

### Standardized Proportional Incidence Ratios (SPIRs) for Malignancy in WS

The excess risk of malignant neoplasms in WS was estimated by SPIR analysis using data from the Japan-resident cases with high WS diagnostic confidence (‘definite’ or ‘probable’ WS diagnoses) and comparison data from the Osaka Japan prefecture (Table 4). SPIRs were strongly elevated for melanomas of the skin (SPIR = 59.7, 95% CI: 27.3, 113.3), soft tissue malignancies (SPIR = 24.3, 95% CI: 12.1, 43.4) and bone malignancies (SPIR = 15.1, 95% CI: 6.9, 28.6). The proportionate incidence of thyroid malignancy was elevated, though to a lesser degree (SPIR = 9.6, 95% CI: 5.2, 16.1). In contrast, there was no elevated risk for leukemia when preleukemic disorders were excluded (SPIR = 1.5, 95% CI: 0.48, 3.5).

**Table 4:** Standardized proportionate incidence ratios (SPIRs) for malignancies in Japan-resident Werner syndrome patients.

site	observed (n = 88)**	expected***	SPIR	95% CI
melanoma of skin	9	0.15	59.7*	(27.3, 113.3)
soft tissue	11	0.45	24.3*	(12.1, 43.4)
bone	9	0.60	15.1*	(6.9, 28.6)
thyroid	14	1.46	9.6*	(5.2, 16.1)
leukemia	5	3.37	1.5	(0.48, 3.46)

* statistically significant result (p<0.05).

** includes WS patients with high WS diagnostic confidence (1965–2009, ages 10–69). Includes benign meningiomas diagnosed prior to 1988, but excludes non-melanoma skin neoplasms.

*** obtained using Osaka, Japan *CI5* volume case data (i.e., representative sample from 1970–2002).

### Standardized Incidence Ratios (SIRs) for Neoplasia in WS

We calculated SIRs as a complementary method to quantify the excess risk of neoplasia in WS. We conducted SIR analyses using WS patients in our cohort with the highest diagnostic confidence and estimated the WS population at risk from the average of reported WS pathogenic allele frequencies (q = 0.0037) [13]. These analyses indicated significantly elevated risk for all six common tumor types observed in Japan-resident WS patients except leukemias, with relative risks ranging from 53.5 (95% CI: 24.5, 101.6) for melanomas of the skin to 8.9 (95% CI: 4.9, 15.0) for thyroid malignancies (see Table 5). We could not further quantify the risk of acral lentiginous melanoma in WS, as there were too few cases in Osaka Cancer Registry data to provide a valid comparison (n = 5, from 1963–2005). The overall risk of malignancy of all types in WS patients was not significantly elevated when compared with an Osaka prefecture reference population (SIR = 0.87, 95% CI: 0.70, 1.07).

**Table 5:** Standardized incidence ratios (SIRs) for malignancies in Japan-resident Werner syndrome patients.***

tumor	observed****	expected****	SIR	95% CI
melanoma of skin	9	0.17	53.5*	(24.5, 101.6)
meningioma	10	0.28	36.2*	(17.3, 66.5)
soft tissue	11	0.35	31.1*	(15.5, 55.7)
bone	9	0.33	27.1*	(12.4, 51.4)
thyroid	14	1.57	8.9*	(4.9, 15.0)
leukemia	5	2.47	2.0	(0.66, 4.7)
all sites**	90	103.81	0.87	(0.70, 1.07)

* statistically significant result (p<0.05).

** includes benign meningiomas diagnosed prior to 1988, but excludes non-melanoma skin neoplasms.

*** for patients with high WS diagnostic confidence (1965–2009).

**** relative to Osaka, Japan population, 1965–2009.

**note:** analysis conditioned on a *WRN* pathogenic allele frequency of *q = 0.0037.*

We performed SIR sensitivity analyses to determine how risk estimates were influenced by pathogenic *WRN* allele frequency (Table S6). Neoplasm type-specific SIRs calculated using the highest estimated pathogenic *WRN* allele frequency (q = 0.006) [12] were 2.5-fold lower than estimates using an average allele frequency (see above), but still significantly increased for melanomas of the skin (SIR = 20.4, 95% CI: 9.3, 38.6), benign or malignant meningioma (SIR = 13.8, 95%: 6.6, 25.3), soft tissue malignancies (SIR = 11.8, 95% CI: 5.9, 21.2), bone malignancies (SIR = 10.3, 95% CI: 4.7, 19.5) and thyroid malignancies (SIR = 3.4, 95% CI: 1.9, 5.7) (Table S6). We did not detect an increase in the risk of leukemia associated with WS (SIR = 0.77, 95% CI: 0.25, 1.8) or malignancies of all types (SIR = 0.33, 95% CI: 0.27, 0.41). Risk analyses performed using the lowest estimated *WRN* pathogenic allele frequency (q = 0.0014) [13] indicated a 7-fold increase in risk across malignancies of all types compared to average allele frequency estimates (see above). These altered risk estimates directly reflect differences in the estimated population of WS patients at risk of neoplasia. Sensitivity analyses conducted to determine the influence of the age distribution of WS patients on neoplasm type-specific risk provided similar relative risk estimates (Table S7), indicating that risk estimates are robust to the estimation of the ages of the WS patient population at risk.

## Discussion

Our goal in this study was to provide a quantitatively rigorous analysis of the spectrum and excess risk of specific neoplasms in WS, a recessive familial cancer predisposition and progeroid syndrome. In order to perform these analyses we assembled a new study population of 189 patients with 248 neoplasms that were well-documented, and in whom we could verify the diagnosis of WS using standardized diagnostic criteria (Table 1). A majority of our study population consisted of WS patients residing in Japan (n = 139, or 74% of patients; Table 3). This is due to both the high prevalence of WS in Japan, and to the persistence of medical journals in Japan that publish case-based research and clinical observations [2], [5]. Additional patients were from a wide diversity of locations outside Japan. The large Japan-resident portion of cases allowed us to calculate neoplasm-specific, as well as overall relative risk of neoplasia by using Japanese population reference data for comparisons. The ratio of males to females in our study population was higher among Japan-resident WS patients than among patients residing outside Japan (79∶58 vs. 23∶26, respectively). The reasons for this are not clear, but prior reports have not identified a gender bias in WS [1], [2] and the apparent difference noted above was not significant (p = 0.24, as determined by a two-tailed Fisher’s exact test).

The large number of uncommon neoplasms (Figure 2) and multiple neoplasms (42/189 patients, or 22%; Table S5) in our study population both identify WS as a heritable cancer predisposition syndrome. Two-thirds (67%) of the neoplasms in our study population patients were of six major histopathologic types (Figure 2). We observed the previously reported difference in the frequency of thyroid neoplasms and, to a lesser degree melanomas, between Japan-resident WS patients and WS patients residing elsewhere [5]. However, these frequency differences were again not statistically significant after correcting for multiple testing (Table S3). As noted previously [14]–[16], geographic differences in thyroid neoplasms and melanomas cannot be explained by differences in the frequency of specific *WRN* mutant alleles, as many of the same pathogenic mutations have been found in WS patients residing in Japan or elsewhere.

Our study provides the first comprehensive analysis of neoplasm type-specific excess risk in WS that makes use of both population-based cancer incidence data and SPIR and SIR analyses (Tables 4 and 5). Leukemia risk was not consistently elevated in all analyses when leukemia alone was considered. However, we suspect that the risk of leukemia and associated hematologic disorders is significantly elevated in WS, as there have been many reports of leukemia and preleukemic disorders (e.g., myelodysplasia) in WS patients (Figure 2). This elevated risk may be related to the age-dependent increase in genetic instability in the peripheral blood in WS patients that we and others have documented [17]–[19].

Our case-based study has several potential limitations. Not all of our case reports unambiguously detailed patient data such as age at diagnosis of neoplasia or the histopathologic information required for our tumor type-specific analyses. However, many of these problems could be resolved by combining available data and applying common decision criteria. For example, we used widely accepted, contemporary diagnostic criteria for WS and for diagnostic confidence levels, and applied these uniformly across all patients (see Table S1). Multiple case reports of the same patient or neoplasm were often useful for filling in detail missing from single reports. We were, however, careful to exclude multiple reports of the same patient to avoid over-counting and over-estimating risk. Case reports that could not be used for our primary analyses were cataloged, as were reports that could not be used for risk estimation analyses due to missing data (Tables S1 and S2).

We also used complementary approaches and sensitivity analyses to assess the potential consequences of uncertainty or bias. For example, WS is underdiagnosed and underreported by virtue of having an incompletely penetrant phenotype of delayed onset [1], [2], [20]. This may result both in low estimates of risk of neoplasia, and a failure to report instances of common neoplasms in a patient prior to a diagnosis of WS. Despite this, our tumor spectrum analysis indicates that we are capturing a broad spectrum of neoplasms in WS including both common and rarer histopathologic types (Figure 2). We were able to more formally address the potential consequences of underreporting on risk by using the SIR as a risk measure. Underreporting of a given tumor type in SIR analyses biases the risk estimate for that tumor type toward the null hypothesis (i.e., no increase in risk). Despite this potential bias, our SIR analyses revealed consistent, statistically significant *elevated* risks for the tumors most commonly reported in WS. In similar fashion, sensitivity analyses performed to address uncertainties in the number and age distribution of WS patients in Japan (Tables S6 and S7) also revealed statistically significant elevations in risk for these tumors. Thus we think our estimates of elevated, type-specific risk of neoplasia in WS are likely to be not only robust but conservative.

One likely cause of the elevated risk of neoplasia in WS is the persistent, constitutional genetic instability and DNA damage sensitivity that are key cellular hallmarks of WS [4], [16]. Treatment of a first primary neoplasm could, as a result, be contributing to the elevated risk of multiple neoplasia in WS patients, but there are few well-documented instances of therapy-induced neoplasms in WS (see Table S5, Case 33 and associated reference for one potential example). There is, however, a report of heightened sensitivity to chemotherapy in a WS patient leading to unintended, therapy-related toxicity and death [21].

The spectrum of neoplasia in WS is similar to but distinct from two other RECQ helicase deficiency syndromes, Bloom syndrome and Rothmund-Thomson syndrome. Bloom syndrome (BS) confers a very strong predisposition to a wide range of neoplasms [22], [23], while in Rothmund-Thomson syndrome (RTS) and related cancer predisposition syndromes resulting from *RECQL4* mutations the cancer predisposition is largely limited to osteosarcomas and lymphomas [24], [25]. The median ages at cancer diagnosis are lower in BS and RTS compared with WS, and the median ages at cancer diagnosis in all three RECQ helicase deficiency syndromes are lower than population median ages at diagnosis for cancers of the same type in the general population (Table 6). The spectrum of neoplasms observed in WS can be compared with other recessive familial cancer predisposition syndromes associated with genetic instability such as Li-Fraumeni syndrome [26], [27]; the heritable bone marrow failure/cancer predisposition syndromes Fanconi anemia [28], [29], dyskeratosis congenita [30], Diamond-Blackfan anemia [31] and Shwachman-Diamond syndrome [32], [33]; and the heritable mismatch repair deficiency syndromes (e.g., homozygous mutations in *MLH1* or *PMS2*) [34]. A better understanding of the molecular mechanisms responsible for predisposing certain cell types and tissues to genetic instability-associated cancer may reveal commonalities among these seemingly disparate diseases, and shared molecular targets or pathways in the respective targeted cell or tissue types.

**Table 6:** Median ages and age ranges for cancer in Werner syndrome versus other cancer predisposition syndromes.

neoplasm*	Werner syndrome**(n = 146)		Bloom syndrome*** (n = 104)	Rothmund-Thomson syndrome**** (n = 19)	Osaka Cancer Registry (n = 2,828)	Osaka population***** (n = 785,798)	US SEER data
melanoma of skin	44 (34, 59)	0		–	60 (2, 96)	62 (2, 85+)	61.0
meningioma	39 (22, 67)	9		–	61 (0, 99)	–	–
soft tissue	43 (26, 58)	0		–	na	52 (2, 85+)	58.0
bone	49 (20, 57)	(4, 15)		12 (3, 31)	na	47 (2, 85+)	41.0
thyroid	40 (25, 46)	0		–	na	57 (2, 85+)	50.0
leukemia	45 (28, 62)	15 (2, 39)		–	na	52 (2, 85+)	66.0
all sites excluding skin	44 (20, 82)	24 (0, 48)		12 (2, 31)	na	67 (2, 85+)	–
**all sites**	**44.5 (20, 82)**	**25 (0, 48)**		**12 (2, 31)**	**na**	**67 (2, 85+)**	**66.0**

* malignant cases only, except for meningiomas where benign cases were also included.

** Japan-residents cases only, excluding tumor cases with ambiguous age at diagnosis.

*** BS = Bloom syndrome. Some discrepancies between data given in Figure 2 and Table 4 of reference [23].

**** RTS = Rothmund-Thomson syndrome [25]. Note that adding 6 additional tumor cases reported in patients with RAPADILINO syndrome, which is also caused by mutations in *RecQL4*, has minimal effect (all sites estimate becomes 13 (2, 33)).

***** Osaka population ages given by 5-year age groups, so medians are approximate. Data obtained from *CI5*plus online application (years 1963–2002); soft tissue and all sites data from *CI5* volumes 3–9 (years 1970–2002) [7]. SEER data from [35].

na = not accessed.

Our analysis of cancer spectrum and risk may provide practical guidance in caring for patients with WS, most notably if cancer surveillance is considered. Our results may also serve to guide genomic and population-based analyses of cancer in *WRN* mutation carriers, and in sporadic cancers of the types most frequently observed in WS. These types of additional analyses have the potential to provide insight into the origins of cancer in WS, and the potential contribution of *WRN* mutations to cancer risk in the general population.

## Supporting Information

Figure S1 PRISMA Checklist.
(DOC)

Figure S2 PRISMA Flow Diagram.
(DOC)

Table S1 Study population spreadsheet (see Excel spreadsheet S1).
(XLSX)

Table S2 Additional case reports not included in the study population (see Excel spreadsheet S2).
(XLSX)

Table S3 Distribution of neoplasm types in Werner syndrome patients residing in Japan versus patients residing outside of Japan (1939–2011).
(DOCX)

Table S4 Japan-resident WS patient thyroid histopathologic subtype analysis.
(DOCX)

Table S5 Multiple primary neoplasms in Werner syndrome patients.
(DOCX)

Table S6 SIR sensitivity analysis conditioned on *WRN* pathogenic allele frequency.
(DOCX)

Table S7 SIR sensitivity analysis conditioned on WS patient age distribution.
(DOCX)

Methods S1 (DOCX)
