# Supplementary material for: Spectrum and Risk of Neoplasia in Werner Syndrome: A Systematic Review
Source: PLoS One. 2013 Apr 1;8(4):e59709. doi: 10.1371/journal.pone.0059709 (PMC3613408; doi:10.1371/journal.pone.0059709)
Supplement: Figure S2 — PRISMA Flow Diagram. (DOC) [file pone.0059709.s002.doc]

**Figure S2: Study PRISMA Flow Diagram**

**Screening**

**Included**

**Eligibility**

**Identification**

Records identified through database searching
(n = 474)

Additional records identified through other sources
(n = 224)

Total records
(n = 698)

Full-text articles assessed for eligibility
(n = 248)

Full-text articles with no tumor listed
(n = 46)

Articles with one or more tumor listed
(n = 202)

Records after duplicates between database searching and other sources removed
(n = 657)

Duplicate records
(n = 41)

Neoplasms in study population
(n = 189 cases, n = 248 neoplasms)

Neoplasms excluded from study population
(n = 80 cases, n = 87 neoplasms)
