# Supplementary material for: Spectrum and Risk of Neoplasia in Werner Syndrome: A Systematic Review
Source: PLoS One. 2013 Apr 1;8(4):e59709. doi: 10.1371/journal.pone.0059709 (PMC3613408; doi:10.1371/journal.pone.0059709)
Supplement: Table S3 — Distribution of neoplasm types in Werner syndrome patients residing in Japan versus patients residing outside of Japan (1939–2011). (DOCX) [file pone.0059709.s005.docx]

**Table S3: Distribution of neoplasm types between WS patients residing in Japan vs. WS patients residing outside of Japan (1939-2011)**

|  | **WS cases residing in Japan** | **WS cases residing outside of Japan** | **p-value (Fisher’s exact)** |
| --- | --- | --- | --- |
| **neoplasm type** |  |  |  |
| soft tissue | 20 | 5 | 0.63 |
| bone | 13 | 6 | 0.59 |
| melanomas | 29 | 4 | 0.057 |
| hematologic/lymphoid | 17 | 6 | 1.0 |
| thyroid | 36 | 4 | 0.011 |
| meningioma | 17 | 10 | 0.17 |
| other | 52 | 29 |  |
| **total** | **184** | **64** | **0.014*** |

*indicates significant at α=0.05.
