# Supplementary material for: Spectrum and Risk of Neoplasia in Werner Syndrome: A Systematic Review
Source: PLoS One. 2013 Apr 1;8(4):e59709. doi: 10.1371/journal.pone.0059709 (PMC3613408; doi:10.1371/journal.pone.0059709)
Supplement: Table S4 — Japan-resident WS patient thyroid histopathologic subtype analysis. (DOCX) [file pone.0059709.s006.docx]

**Table S4: Japan-resident WS patient thyroid histopathologic subtype analysis**

| **Thyroid cancer subtype** | **WS cases** | **Osaka population comparison data*** | |
| --- | --- | --- | --- |
|  |  | ages 0-24 | ages 10-69 |
| follicular subtype | 11 | 14 | 402 |
| papillary subtype | 7 | 102 | 2,391 |
| other/unspecified type | 5 | 29 | 583 |
| **Total** | **23** | **145** | **3,376** |
| *p –value (Fisher’s Exact)* |  | *p=0.00002* | *p=0.00001* |

*comparison data from Osaka, Japan population cases reported in *CI5* (vol. 7-9, 1988-2002).
