# Supplementary material for: Spectrum and Risk of Neoplasia in Werner Syndrome: A Systematic Review
Source: PLoS One. 2013 Apr 1;8(4):e59709. doi: 10.1371/journal.pone.0059709 (PMC3613408; doi:10.1371/journal.pone.0059709)
Supplement: Table S5 — Multiple primary neoplasms in Werner syndrome patients. (DOCX) [file pone.0059709.s007.docx]

**Table S5: Multiple primary neoplasms in Werner syndrome patients**

| **Case** | **Sex** | **WS Dx confidence** | **Tumor diagnosis – type and site (age at diagnosis)****** | **Ref. #** |
| --- | --- | --- | --- | --- |
| 4 | M | probable | osteosarcoma of left tibia (45), malignant melanoma of left foot, plantar surface (45), gastric adenocarcinoma(45), leiomyosarcoma of lung (45), papillary carcinoma of thyroid* (45) | [1] |
| 6 | M | possible | meningioma (~34), myelofibrosis (41), follicular adenomas of thyroid* (41) | [2] |
| 19 | M | definite | leiomyosarcoma of left bicep (40), follicular adenoma of thyroid* (~40) | [3] |
| 20 | F | definite | transitional cell carcinoma of bladder (38), malignant melanoma of nasal cavity (45) | [4] |
| 23 | F | probable | papillary carcinoma of thyroid (~47), osteosarcoma of right lower leg (~49) | [5] |
| 25 | F | probable | uterine cancer NOS (34), follicular carcinoma of thyroid (36), osteosarcoma of left knee (~37) | [6] |
| 27 | F | probable | malignant melanoma of nasal cavity (~45), malignant melanoma of right foot, 5th toe (46) | [7] |
| 30 | F | unknown | malignant melanoma of: right large pudental lip (~44), left foot, plantar surface , near toe and near heel (~44) | [8] |
| 33 | F | probable | papillary carcinoma of thyroid (~32), AML FAB M4, after RAEB-t/MDS (40) | [9] |
| 46 | F | unknown | nasal cancer NOS (age uk), uterine cancer NOS (age uk) | [10] |
| 49 | M | definite | thyroid neoplasm NOS (27), papillary carcinoma of thyroid (46), meningioma (46) | [11] |
| 64 | M | possible | meningioma (62), MDS (62) | [12] |
| 69** | F | unknown | follicular carcinoma of thyroid (44), thyroid adenoma NOS (44) | [13] |
| 70 | M | definite | thyroid neoplasm NOS (43), malignant peripheral nerve sheath tumor of right knee (~47), undifferentiated pleomorphic sarcoma of right thigh (50), osteosarcoma of right calcaneal region (57) | [14] |
| 76 | F | definite | uterine leiomyoma (29) leiomyoma of left lower abdomen (~34), cystadenocarcinoma of right ovary (37), thyroid adenoma NOS* (37) | [15] |
| 80 | M | probable | thyroid adenoma NOS (48), bronchiolo-alveolar adenocarcinoma of lung (52) | [16] |
| 82 | M | definite | pharyngeal carcinoma NOS (<52), adenocarcinoma of lung (52) | [17] |
| 83 | M | probable | fibrosarcoma of unknown site (~44), meningioma (~44) | [18] |
| 85 | M | possible | SCC of median soft palate (69), SCC of left edge of tongue (74), SCC of right edge of tongue (75), SCC of hard palate (79), SCC of esophagus (79), transitional cell carcinoma of right ureter (82) | [19] |
| 91 | M | probable | follicular carcinoma of thyroid (51), retroperitoneal leiomyosarcoma (51) | [20] |
| 93 | M | unknown | bone neoplasm NOS of femur (38), thyroid carcinoma NOS (39), benign fibrous histiocytoma of femur (53) | [21] |
| 95** | M | definite | glioblastoma (26), thyroid neoplasm NOS (26) | [22] |
| 97 | M | definite | osteosarcoma of right lower leg (50), malignant melanoma of right conjunctiva (52) | [23] |
| 98 | M | unknown | acral lentiginous melanoma *in situ* of right thumb (~21), malignant melanoma of nasal cavity (~31) | [24] |
| 108** | F | probable | papillary carcinoma of thyroid (22), thyroid adenoma NOS (39) | [25] |
| 111 | F | probable | lobular breast carcinoma (26), gastric adenocarcinoma (~28) | [26] |
| 112 | F | probable | thyroid adenoma NOS (35), SCC *in situ* of right thigh (54) | [27] |
| 116 | F | definite | uterine leiomyoma (42), meningioma (42) | [28] |
| 119 | M | definite | meningioma (53), malignant melanoma of nasal mucosa (56) | [29] |
| 132 | F | definite | adrenal cortical carcinoma (50), papillary transitional cell carcinoma of right ureter (50) | [30] |
| 141 | M | unknown | osteosarcoma of right leg (52), meningioma (53) | [31] |
| 142 | F | definite | meningioma (56), benign thyroid neoplasm NOS (56) | [32] |
| 300 | M | probable | follicular thyroid neoplasm NOS (35), malignant melanoma of right heel (42), acral lentiginous melanoma of left foot, plantar surface (44) | [33] |
| 349 | M | definite | follicular/clear cell carcinoma of thyroid (34), AML FAB M6 (erythroleukemia) (34) | [34] |
| 403 | F | definite | follicular adenoma of thyroid (29), neurinoma (31) | [35] |

**Table S5: Multiple primary neoplasms in Werner syndrome patients (cont.)**

| **Case** | **Sex** | **WS Dx confidence** | **Tumor diagnosis – type and site (age at diagnosis)****** | **Ref. #** |
| --- | --- | --- | --- | --- |
| 161 | M | probable | malignant melanoma of finger (<44), AML FAB M2 (44) | [36] |
| 168 | M | probable | basal cell carcinoma on right tip of nose (52), SCC on scalp and left forearm (57) | [37] |
| 171 | uk | unknown | orbital osteosarcoma (age uk), thyroid adenoma NOS (age uk), meningioma* (age uk) | [38] |
| 172 | M | possible | fibrosarcoma of mediastinum (46), basal cell carcinoma on nose (x 2) and on upper lip (57) | [39] |
| 180 | F | probable | undifferentiated pleomorphic sarcoma of left lateral thigh (40), cystadenocarcinoma of ovary (41) | [40] |
| 183 | F | definite | SCC on cheek (52), basal cell carcinoma x 2 on face (52) | [41] |
| 185 | F | definite | breast carcinoma NOS (<47), ovarian carcinoma NOS (<47), pancreatic adenocarcinoma of head of pancreas (47), carcinoid of lung* (47), | INU 1010  *** |
| 186 | M | definite | hepatocellular carcinoma (42), cortical adenoma of adrenal gland* (42) | [42] |
| 188 | F | definite | cholangiocarcinoma (42), meningioma* (42), cortical adenoma of adrenal gland* (42) | [43] |
| 191** | M | possible | acral lentiginous melanoma of left thumb and lentigo maligna melanoma under left ear (56) | [44] |
| 200 | F | probable | meningioma (~31), thyroid carcinoma NOS (~49) | [45] |

*incidental finding at autopsy.

**excluded from multiples count due to the ambiguity in the case reports between thyroid goiter and adenoma or because multiple tumors at the same organ site

***unpublished case #INU1010 from the International Registry of Werner Syndrome (personal communication from G. M. Martin and J. Oshima).

****multiple malignancies of the same histology at the same organ site or multiple benign tumors are counted only once in spectrum/risk estimates.

uk = unknown

MDS = myelodysplasia

SCC = squamous cell carcinoma

NOS = not otherwise specified

AML = acute myelogenous leukemia

FAB = French-American-British classification

**Supplement References**

1. Tsuchiya H, Tomita K, Ohno M, Inaoki M, Kawashima A (1991) Werner's syndrome combined with quintuplicate malignant tumors: a case report and review of literature data. Jpn J Clin Oncol 21: 135-142.

2. Noda M, Matsui K, Kitagawa M, Ohta M (1990) Werner's syndrome with myelofibrosis following diphenylhydantoin therapy after meningiomectomy: an autopsy case. J Am Geriatr Soc 38: 59-61.

3. Shinohara K, Kojima T, Nakagawa S, Ishikawa F, Murakami T (1974) [Autopsy case of Werner's syndrome associated with smooth muscle sarcoma]. Nippon Rinsho 32: 859-865.

4. Yamada H, Okada E, Kim H, Sakakura Y (1991) [A case of Werner's syndrome associated with bladder and nasal malignancies]. Jibi Inkoka Tokeibu Geka 63: 139-143.

5. Ichioka S, Tanaka H, Uchiyama K, Yamada A (1991) [A case of Werner's syndrome associated with double cancer]. Keisei Geka 34: 975-982.

6. Ozawa M, Arita D, Hino M, Ko T, Ishihara T, et al. (1990) [A case of Werner's syndrome associated with uterine cancer, thyroid cancer, and osteosarcoma]. Pharma Medica 8: 137.

7. Uehara T, Sakai Y, Isonokami M, Yamamura T, Okada N, et al. (1994) [A case of Werner's syndrome associated with melanoma of the nasal cavity and toe]. Rinhi 48: 95-98.

8. Shibuya H, Kato A, Kai N, Fujiwara S, Goto M (2005) A case of Werner syndrome with three primary lesions of malignant melanoma. J Dermatol 32: 737-744.

9. Takemoto Y, Hata T, Kamino K, Mitsuda N, Miki T, et al. (1995) Leukemia developing after 131I treatment for thyroid cancer in a patient with Werner's syndrome: molecular and cytogenetic studies. Intern Med 34: 863-867.

10. Mano T, Imamura Y, Mano K, Kawakita S, Tani N, et al. (1990) [A case of Werner's syndrome associated with primary hypothyroidism]. Ronen Igaku 28: 553-556.

11. Akiho H, Takayanagi R, Migita Y, Yamashita T, Nawada S (1993) [A case of Werner's syndrome associated with papillary carcinoma of thyroid and brain tumor]. Rinsho to Kenkyu 70: 3879-3885.

12. Taketomi K, Goto Y, Arai I, Ichinose M, Kinoshita K (1990) [An autopsy case of Werner's syndrome associated with meningioma and myelodysplastic syndrome]. To-Nyo-Byo 33: 180.

13. Takeuchi T, Oki S, Shimazu T, Kubo S, Nakazima K, et al. (1985) [Two cases of Werner's syndrome associated with diabetes mellitus, malignant tumor, and fatty liver]. Nihon Naika Gakkai Zasshi 74: 855-856.

14. Nakamura Y, Shimizu T, Ishikawa Y, Matsumoto T, Sugimoto M, et al. (2003) Triple primary sarcoma in Werner syndrome with a novel mutation. Rheumatology (Oxford) 42: 798-800.

15. Ueno T, Miwa U, Onoe N, Iwaki N, Nakano Y, et al. (1976) [An autopsy case of Werner's syndrome with death from ovarian cancer associated with multiple benign tumors]. Naika 37: 533-537.

16. Yamanaka A, Hirai T, Ohtake Y, Kitagawa M (1997) Lung cancer associated with Werner's syndrome: a case report and review of the literature. Jpn J Clin Oncol 27: 415-418.

17. Ohnishi S, Fujimoto M, Oide T, Nakatani Y, Tsurutani Y, et al. (2010) Primary lung cancer associated with Werner syndrome. Geriatr Gerontol Int 10: 319-323.

18. Nakao Y, Hattori T, Takatsuki K, Kuroda Y, Nakaji T, et al. (1980) Immunological studies on Werner's syndrome. Clin Exp Immunol 42: 10-19.

19. Iguchi H, Takayama M, Kusuki M, Sunami K, Nakamura A, et al. (2004) A possible case of Werner syndrome presenting with multiple cancers. Acta Otolaryngol: 67-70.

20. Takuma E, Naruse R, Arai H, Komatsu K, Morita T, et al. (1988) [A case of Werner's syndrome associated with multiple neoplasms]. Nisseki Igaku 40: 20.

21. Shinjo K, Asai T, Tsuboi S, Takahashi Y, Ichihara S, et al. (1999) [A case of multiple bone tumor complicated by Werner syndrome]. J Tokai Bone Soft Tissue Tumors 10: 17-20.

22. Tonami H, Hamada S, Nishiki T, Yamamoto I, Yamazaki Y, et al. (1983) [Werner's syndrome]. Rinsho Hoshasen 28: 1479-1485.

23. Ichihashi N, Kamiya H, Takagi H, Kitajima Y, Osada K (1997) [A case of Werner's syndrome associated with malignant melanoma and osteosarcoma]. Skin Cancer 12: 366-369.

24. Yoshida T, Inoue Y, Kiyohara Y, Suzuki T, Tsuchida T, et al. (1998) [A case of malignant melanoma of a nasal cavity associated with Werner's syndrome with chemotherapy with intra-arterial infusion]. Skin Cancer 13: 61-65.

25. Murakami T, Tajiri A, Murakami N, Noguchi S (1988) [Abnormal glucose tolerance and lipid metabolism in Werner's syndrome]. Ooitaken Igaku kai Zasshi 7: 71-74.

26. Kurosawa M, Kan M, Naganuma H, Sawai T, Sasano N (1982) [Werner's syndrome associated with breast cancer and gastric cancer]. Igaku no Ayumi 121: 1057-1067.

27. Tamada K, Nakamura Y, Habe K (2000) [A case of Werner's syndrome associated with Bowen disease]. Skin Cancer 15: 258-261.

28. Sugimoto N, Terayama K, Fujioka F, Maeda T (1991) [Four cases of Werner's syndrome with special reference to the orthopedic aspect]. Chubu Nippon Seikei Geka Saigai Geka Gakkai Zasshi 34: 786-790.

29. Uehara S, Koike T, Azegami T, Yamazaki T (2004) [Autopsy of a Werner's Syndrome patient who died from malignant melanoma]. Shinshu Igaku Zasshi 52: 15-20.

30. Takazawa R, Ajima J, Yamauchi A, Goto M (2004) Unusual double primary neoplasia: adrenocortical and ureteral carcinomas in Werner Syndrome. Urol Int 72: 168-170.

31. Tsurubuchi T, Yamamoto T, Tsukada Y, Matsuda M, Nakai K, et al. (2008) Meningioma associated with Werner syndrome--case report. Neurol Med Chir (Tokyo) 48: 470-473.

32. Nakamura Y, Shimizu T, Ohigashi Y, Itou N, Ishikawa Y (2005) Meningioma arising in Werner syndrome confirmed by mutation analysis. J Clin Neurosci 12: 503-506.

33. Kobori S, Seno J, Kato H, Kitajima Y, Sakamoto Y (1987) [A case of Werner's syndrome associated with malignant melanoma]. Rinsho Hifuka 29: 691-696.

34. Maruyama M, Kaneko K, Kurokawa I, Arai O (1987) [A case of Werner Syndrome associated with thyroid cancer and erythroid leukemia]. Nihon Naika Gakkai Zasshi 76: 762-763.

35. Matsuda F, Miyazaki K, Nakai T, Iwase H, Saito T (1983) [A case of Werner's syndrome with neurinoma in the thoracolumbar region]. Seikei Geka 34: 85-90.

36. Seiter K, Qureshi A, Liu D, Galvin-Parton P, Arshad M, et al. (2005) Severe toxicity following induction chemotherapy for acute myelogenous leukemia in a patient with Werner's syndrome. Leuk Lymphoma 46: 1091-1095.

37. Zalla JA (1980) Werner's syndrome. Cutis 25: 275-278.

38. Jacobson HG, Rifkin H, Zucker-Franklin D (1960) Werner's syndrome: a clinical-roentgen entity. Radiology 74: 373-385.

39. Hrabko RP, Milgrom H, Schwartz RA (1982) Werner's syndrome with associated malignant neoplasms. Arch Dermatol 118: 106-108.

40. Bjornberg A (1976) Werner's syndrome and malignancy. Acta Derm Venereol 56: 149-150.

41. Cerimele D, Cottoni F, Scappaticci S, Rabbiosi G, Borroni G, et al. (1982) High prevalence of Werner's syndrome in Sardinia: description of six patients and estimate of the gene frequency. Hum Genet 62: 25-30.

42. Oppenheimer BS, Kugel VH (1941) Werner's syndrome: report of the first necropsy and of findings in a new case. Am J Med Sci 202: 629-642.

43. Rogers A (1959) Werner's syndrome: report of case with unusual complication. J Fla Med Assoc 46: 436-438.

44. Barnett JH, Lee FG, Rinsky MJ (1983) Acral lentiginous melanoma and lentigo maligna occurring in Werner's syndrome. Cutis 32: 277-283.

45. Runne U (1975) [Werner's syndrome with metastasizing malignant struma]. Z Hautkrankheiten 50: 817-819.
