# Supplementary material for: Spectrum and Risk of Neoplasia in Werner Syndrome: A Systematic Review
Source: PLoS One. 2013 Apr 1;8(4):e59709. doi: 10.1371/journal.pone.0059709 (PMC3613408; doi:10.1371/journal.pone.0059709)
Supplement: Table S6 — SIR sensitivity analysis conditioned on WRN pathogenic allele frequency. (DOCX) [file pone.0059709.s008.docx]

**Table S6: SIR sensitivity analysis conditioned on *WRN* pathogenic allele frequency in Japan-resident Werner syndrome patients with high diagnostic confidence**

***Table S6A: SIR sensitivity analysis conditioned on a WRN pathogenic allele frequency of q=0.006***

| **neoplasm** | **observed** | **expected**** | **SIR** | **95% CI** |
| --- | --- | --- | --- | --- |
| melanoma of skin | 9 | 0.44 | 20.4* | (9.3, 38.6) |
| meningioma | 10 | 0.73 | 13.8* | (6.6, 25.3) |
| soft tissue | 11 | 0.93 | 11.8* | (5.9, 21.2) |
| bone | 9 | 0.87 | 10.3* | (4.7, 19.5) |
| thyroid | 14 | 4.12 | 3.4* | (1.9, 5.7) |
| leukemia | 5 | 6.50 | 0.77 | (0.25, 1.8) |
| **all sites***** | 90 | 273.0 | 0.33* | (0.27, 0.41) |

*statistically significant result (p<0.05).

**relative to Osaka, Japan population, 1965-2009.

***includes benign meningiomas diagnosed prior to 1988, but excludes non-melanoma skin neoplasms.

***Table*** ***S6B: SIR sensitivity analysis conditioned on a WRN pathogenic allele frequency of q=0.0014***

| **neoplasm** | **observed** | **expected**** | **SIR** | **95% CI** |
| --- | --- | --- | --- | --- |
| melanoma of skin | 9 | 0.02 | 373.9* | (171.0, 709.7) |
| meningioma | 10 | 0.04 | 252.6* | (121.1, 464.6) |
| soft tissue | 11 | 0.05 | 217.5* | (108.6, 389.2) |
| bone | 9 | 0.05 | 189.0* | (86.4, 358.8) |
| thyroid | 14 | 0.22 | 62.4* | (34.1, 104.7) |
| leukemia | 5 | 0.35 | 14.1* | (4.6, 33.0) |
| **all sites***** | 90 | 14.9 | 6.1* | (4.9, 7.4) |

*statistically significant result (p<0.05).

**relative to Osaka, Japan population, 1965-2009.

***includes benign meningiomas diagnosed prior to 1988, but excludes non-melanoma skin neoplasms.
