# Supplementary material for: Spectrum and Risk of Neoplasia in Werner Syndrome: A Systematic Review
Source: PLoS One. 2013 Apr 1;8(4):e59709. doi: 10.1371/journal.pone.0059709 (PMC3613408; doi:10.1371/journal.pone.0059709)
Supplement: Table S7 — SIR sensitivity analysis conditioned on WS patient age distribution. (DOCX) [file pone.0059709.s009.docx]

**Table S7: SIR sensitivity analysis conditioned on WS patient age distribution in Japan-resident Werner syndrome patients with high diagnostic confidence**

***Table S7A: SIR sensitivity analysis with age contraction beginning at age 40 years and a WRN pathogenic allele frequency of q=0.0037***

| **neoplasm** | **observed** | **expected**** | **SIR** | **95% CI** |
| --- | --- | --- | --- | --- |
| melanoma of skin | 9 | 0.19 | 46.2* | (21.1, 87.7) |
| meningioma | 10 | 0.33 | 30.6* | (14.7, 56.2) |
| soft tissue | 11 | 0.40 | 27.6* | (13.8, 49.4) |
| bone | 9 | 0.36 | 24.8* | (11.3, 47.0) |
| thyroid | 14 | 1.76 | 8.0* | (4.4, 13.4) |
| leukemia | 5 | 2.75 | 1.8 | (0.59, 4.2) |
| **all sites***** | 90 | 131.53 | 0.68* | (0.55, 0.84) |

*statistically significant result (p<0.05).

**relative to Osaka, Japan population, 1965-2009.

***includes benign meningiomas diagnosed prior to 1988, but excludes non-melanoma skin neoplasms.

***Table S7B: SIR sensitivity analysis with age contraction beginning at age 20 years and a WRN pathogenic allele frequency of q=0.0037***

| **neoplasm** | **observed** | **expected**** | **SIR** | **95% CI** |
| --- | --- | --- | --- | --- |
| melanoma of skin | 9 | 0.14 | 62.6* | (28.6, 118.9) |
| meningioma | 10 | 0.23 | 44.3* | (21.2, 81.5) |
| soft tissue | 11 | 0.31 | 35.1* | (17.5, 62.9) |
| bone | 9 | 0.31 | 29.0* | (13.2, 55.0) |
| thyroid | 14 | 1.35 | 10.3* | (5.7, 17.4) |
| leukemia | 5 | 2.19 | 2.3 | (0.74, 5.3) |
| **all sites***** | 90 | 79.01 | 1.1 | (0.92, 1.4) |

*statistically significant result (p<0.05).

**relative to Osaka, Japan population, 1965-2009.

***includes benign meningiomas diagnosed prior to 1988, but excludes non-melanoma skin neoplasms.
