# Supplementary material for: Spectrum and Risk of Neoplasia in Werner Syndrome: A Systematic Review
Source: PLoS One. 2013 Apr 1;8(4):e59709. doi: 10.1371/journal.pone.0059709 (PMC3613408; doi:10.1371/journal.pone.0059709)
Supplement: Methods S1 — (DOCX) [file pone.0059709.s010.docx]

**Methods S1:**

***WS case-finding and documentation:*** WS patients with neoplasia in Japan were identified by searching J-EAST (http://sciencelinks.jp/j-east/) using the search terms <“Werner” OR “Werner’s” AND “syndrome”>. Additional case reports of individuals with neoplasia from Japan were identified by searching PubMed (http://www.ncbi.nlm.nih.gov/pubmed/) using the following search terms:<"werner syndrome"[MeSH Terms] OR ("werner"[All Fields] AND "syndrome"[All Fields]) OR "werner syndrome"[All Fields] OR ("werner's"[All Fields] AND "syndrome"[All Fields]) OR "werner's syndrome"[All Fields]) AND ("case reports"[Publication Type] OR "case report"[All Fields])>.

To identify WS patients with neoplasms residing outside of Japan, we conducted searches in PubMed and Google Scholar (http://scholar.google.com/) using the search terms <‘Werner’>, <‘Werner’s’> and <‘Syndrome’> and <‘Werner’s syndrome case report’> in conjunction with <‘cancer’>, <‘tumor’> and <‘neoplasm’>. Articles were reviewed in full if they referred to WS and noted a tumor or neoplasm in the title or abstract. Additional reports of neoplasia in WS were identified by reviewing WS case reports that did not specifically refer to a ‘tumor,’ ‘neoplasm’ or ‘cancer’ in the title or abstract. The reports assembled from these searches were cross-correlated with previously published case reports or series (see, e.g., Goto *et al*. 1996) [1] to identify and remove duplicate reporting. We also requested information on WS patients with neoplasia from the International Registry of Werner Syndrome (http://www.path-ology.washington.edu/research/werner/registry/registry.html), based at the University of Washington. The Registry was founded in 1992 by Dr. George M. Martin and colleagues to establish consistent diagnostic criteria for, and further promote the study of, WS. The current director is Dr. Junko Oshima. The Registry focus since cloning of the *WRN* gene in 1996 has been on the molecular diagnosis of Werner syndrome and related disorders. A locus-specific mutational database for *WRN* was built by one of us (R.J.M, Jr.) and is included as a link (http://www.pathology.washington.edu/research/werner/database/) from the Registry homepage. Registry case files include several patients with neoplasia (see, e.g., Huang *et al.* 2006 [9]), but only a small subset of these are newly reported and/or well-enough documented to be included in our case series (see, *e.g.*, case #154 in Table S1: Study population spreadsheet and case #185 in Table S5 below).

We verified the clinical diagnosis of Werner syndrome in all reported cases using diagnostic criteria established by the International Registry of Werner Syndrome (http://www.wernersyndrome.org/registry/diagnostic.html). All subjects were assigned as well a diagnosis of ‘definite’, ‘probable’, ‘possible’ or ‘uncertain’ WS (Table 1, Table S1: Study population spreadsheet). We also collected patient-specific and tumor-specific information: gender, cause and age at death where specified, and neoplasm-specific data including year of tumor diagnosis (when unspecified, the year prior to the year of publication was substituted) and age at tumor diagnosis, pathologic diagnosis, a diagnosis of malignancy, anatomic site(s), whether a finding was incidental at autopsy, and whether descriptions or images of gross pathology and histopathology were provided (Table S1: Study population spreadsheet ). These data were collected independently by three of the authors (J.M.L, A.K. or R.J.M., Jr.). Tumor diagnoses were verified by comparing the reported diagnosis with patient-specific gross pathology and histopathology images whenever available by one of the authors (R.J.M, Jr.).

Recurrent and multiple neoplasms of the same type at the same site were not counted as independent primary neoplasms following previously established guidelines for reporting multiple primary neoplasms [2]. Anecdotal or personal observations or communications of tumors by authors of published articles were eligible for inclusion as primary case reports, and data from multiple reports of the same patient were pooled whenever possible to improve data completeness (see cases with multiple references in Table S1: Study population spreadsheet). Tumor and patient data were used to identify specific patients and neoplasms to avoid multiple counting of these in our final study population (reviewed by J.M.L., A.K. and R.J.M, Jr.). We excluded tumor reports from this study population which reported an unspecified malignancy (could not be assigned to ICD-10 C00-96 but C44); lacked patient-specific clinical or demographic data; could not be traced to a referenced primary case report describing the patient or neoplasm; or duplicated reports already included in the study population.

***SPIR and SIR as measures of cancer risk:*** SPIRs provide a measure of the over-representation of a particular disease type in a cohort when incident cases are collected, but the size of the underlying population at risk is not defined [3]. However, SPIR estimates may be biased if case ascertainment is biased. For example, cancer types that are rare in the general population are more likely to be reported, especially in the context of a syndrome such as WS. SIR estimates, in contrast, are not affected by under-reporting of other cancers but require estimating the population size, age and gender distribution of individuals at risk.

***Standardized proportionate incidence ratios (SPIRs) for malignancy in WS:*** Each proportion was calculated as the fraction of all malignancies observed, except for non-melanoma skin cancers (ICD-10 C00-96 but C44). We excluded the following neoplasms reported in WS patients from this analysis and the SIR analysis (see below): non-malignant tumors (except meningiomas, which are included in ‘ICD-10 C00-96 but C44’ counts prior to 1988); incidental findings at autopsy; non-melanoma skin cancers (ICD-10 C44); tumors reported without an age at tumor diagnosis (to prevent multiple counting of the same tumor); and tumors diagnosed after 2009. Proportions were compared using indirect standardization [4].

***Standardized incidence ratios (SIRs) for malignancy in WS:*** The SIR was calculated as the ratio of the total number of WS tumor cases of a specific tumor type observed from 1965-2009 to the expected number. To obtain the expected number, we applied population cancer incidence rates to the estimated number of WS patients in the Japan population. Annual estimates of the number of WS patients in Japan were calculated from frequency estimates of pathogenic *WRN* alleles in Japan (q=0.0014 to q=0.006) [5], [6], and the total population size using the Hardy-Weinberg equilibrium: n_WS_ = n_pop_*q^2^, where

q = the frequency of *WRN* pathogenic alleles in the population

n_WS_ = number of WS patients

n_pop_ = population size

For initial estimates we used the average of pathogenic allele frequency estimates (i.e., q=0.0037), and data from the 1965-2009 annual Japan population estimates by the Statistics Bureau of Japan [7].

In order to estimate the age distribution of WS patients, we used Japan population data that included population size estimates by gender and 5-year age grouping between 1965 and 2009 (see above). We adjusted the age distribution of the general population to account for the shortened life expectancy in WS, where the median age at death is 54.3 years [8], [9], then estimated the WS patient population at risk by age interval. We assumed the proportion of the WS population age 0-29 years would be the same as the proportion of the general Japan population age 0-29 years, and thereafter assumed each 15-year age group in the general population up to age 74 years was equivalent to a 10-year age group in WS (e.g., general population ages 30-44 years was equivalent to WS patient ages 30-39 years). At the upper age range, the proportion of individuals ages 75+ years in the general population was considered equivalent to the proportion of WS patients at ages 60+ years. This method accounts for trends in Japanese birth and death rates over time. For sensitivity analyses, we generated alternate WS patient population distributions by changing the earliest age at which we assume the general population age distribution differs from the WS population age distribution (i.e., at age 20 years or 40 years).

Osaka prefecture neoplasm-specific population incidence data were obtained from *CI5* data, in which annual cases were categorized by gender and divided into 5-year age groups. CI5*plus* online (http://ci5.iarc.fr/CI5plus/ci5plus.htm, see Ferlay et al. 2010) [10] was used to obtain annual case numbers for years 1965-2002 for: bone malignancies (ICD-10 C40-41), malignant melanomas of the skin (ICD-10 C43), leukemias (ICD-10 C91-95), thyroid malignancies (ICD-10 C73), and all sites but non-melanoma skin (ICD-10 C00-96 but C44). Because CI5*plus* online does not provide annual case numbers for soft and connective tissue malignancies (ICD-10 C47 & C49) or for all GI malignancies (ICD-10 C15-25), we obtained these data from *CI5* volumes for years 1970-2002. Additionally, case data on meningeal neoplasms (ICD-10 codes C70.0-70.9, D32.0-32.9, D42.0-42.9) categorized by gender and divided into 5-year age groupings were obtained from the Osaka Cancer Registry. Incidence rates were calculated using case numbers and annual Osaka population estimates categorized by gender and divided into 5-year age groups as provided by the Osaka Cancer Registry. Available incidence rates were applied to any years with missing data (e.g., reported incidence rates from 1970-1971 were applied to 1965-1969 to obtain the likely expected case numbers within that time period).

**Supplement References**

1. Goto M, Miller RW, Ishikawa Y, Sugano H (1996) Excess of rare cancers in Werner syndrome (adult progeria). Cancer Epidemiol Biomarkers Prev 5: 239-246.

2. Fritz A, Percy C, Jack A, Shanmugaratnam K, Sobin L, et al., editors (2000) International Classification of Diseases for Oncology. 3rd ed. Geneva: World Health Organization.

3. Breslow NE, Day NE (1987) Proportional measures of incidence and mortality. Statistical Methods in Cancer Research: Vol II--The design and analysis of cohort studies. Lyon: IARC. pp. 76-79.

4. Koepsell TD, Weiss NS (2003) Confounding and Its Control. Epidemiologic Methods: Studying the Occurence of Illness. New York: Oxford UP. pp. 247-280.

5. Schellenberg GD, Miki T, Yu CE, Nakura J (2001) Werner syndrome. In: Valle D, Beaudet AL, Vogelstein B, Kinzler KW, Antonarakis SE et al., editors. Online Metabolic and Molecular Basis of Inherited Disease. New York: McGraw-Hill. pp. 1-36.

6. Satoh M, Imai M, Sugimoto M, Goto M, Furuichi Y (1999) Prevalence of Werner's syndrome heterozygotes in Japan. Lancet 353: 1766.

7. Statistics Bureau of Japan Historical Statistics of Japan: Population and Households. Ministry of Internal Affairs and Communications. http://www.stat.go.jp/english/data/chouki/02.htm. Accessed 2012 Apr 19.

8. Goto M (1997) Hierarchical deterioration of body systems in Werner's syndrome: implications for normal ageing. Mech Ageing Dev 98: 239-254.

9. Huang S, Lee L, Hanson NB, Lenaerts C, Hoehn H, et al. (2006) The spectrum of WRN mutations in Werner syndrome patients. Hum Mutat 27: 558-567.

10. Ferlay J, Parkin DM, Curado MP, Bray F, Edwards B, et al. (2010) Cancer Incidence in Five Continents, Volumes I to IX website. Lyon, France: International Agency for Research on Cancer. http://ci5.iarc.fr. Accessed 2012 Jun 19.
